# Determination of the Relative Efficacy of Eicosapentaenoic Acid and Docosahexaenoic Acid for Anti-Cancer Effects in Human Breast Cancer Models

**DOI:** 10.3390/ijms18122607

**Published:** 2017-12-04

**Authors:** Laura VanderSluis, Vera C. Mazurak, Sambasivarao Damaraju, Catherine J. Field

**Affiliations:** 1 Department of Agricultural, Food and Nutritional Science, Faculty of Agricultural, Life and Environmental Sciences, Li Ka Shing Center for Health Research Innovation, University of Alberta, Edmonton, AB T6G 2E1, Canada; lvanders@ualberta.ca (L.V.); vmazurak@ualberta.ca (V.C.M.); 2 Department of Laboratory Medicine and Pathology, Faculty of Medicine and Dentistry, University of Alberta, Edmonton, AB T6G 2R3, Canada; sdamaraj@ualberta.ca

**Keywords:** docosahexaenoic acid, eicosapentaenoic acid, epidermal growth factor receptor, Triple Negative Breast Cancer, ER+, HER2+, membranes, lipid rafts

## Abstract

Epidemiological studies have associated high fish oil consumption with decreased risk of breast cancer (BC). n-3 long chain polyunsaturated fatty acids (n-3 LCPUFA), eicosapentaenoic acid (EPA) and docosahexaenoic acid (DHA) found in fish and fish oils exert anti-cancer effects. However, few studies have examined the relative efficacy of EPA and DHA alone and in mixtures on BC subtypes. This was the objective of the present review, as this research is a necessity for the translation of findings to human health and disease. The literature suggests that DHA has a greater anti-cancer effect in triple negative BC (TNBC). In estrogen positive (ER+) BC, DHA has a greater effect on cell viability, while both fatty acids have similar effects on apoptosis and proliferation. These effects are associated with preferential uptake of DHA into TNBC lipid rafts and EPA in ER+ BC. EPA:DHA mixtures have anti-cancer activity; however, the ratio of EPA:DHA does not predict the relative incorporation of these two fatty acids into membrane lipids as EPA appears to be preferentially incorporated. In summary, DHA and EPA should be considered separately in the context of BC prevention. The elucidation of optimal EPA:DHA ratios will be important for designing targeted n-3 LCPUFA treatments.

## 1. Introduction

Breast cancer (BC) is the most commonly diagnosed form of cancer in North American women [1] and accounts for 26% of new diagnoses in Canadian women [2]. Fish and fish oil supplements are sources of the 20 and 22 hydrocarbon n-3 long chain polyunsaturated fatty acids (n-3 LCPUFA) eicosapentaenoic acid (EPA) and docosahexaenoic acid (DHA) [3]. Epidemiological evidence has found that increased consumption of EPA and DHA from fish oil is associated with a decreased risk of BC [4,5]; however, a meta-analysis of 21 prospective studies concluded that there was insufficient evidence for an association with fish intake and BC risk [4]. EPA and DHA have anti-cancer effects in human BC cell lines and rodents implanted with established mammary tumours or human BC cells. BC is a heterogeneous disease that can be classified into five different subtypes based on the expression of estrogen (ER), progesterone (PR), and human epidermal growth factor 2 (HER2) receptors related to cell growth and survival [6]. Researchers use different human BC cell lines to represent these subtypes, which vary in aggressiveness and prognosis [6]. The mechanisms responsible for anti-cancer effects are multiple and considering all n-3 LCPUFA as acting the same across BC subtypes could limit the effective translation of targeted dietary recommendations for BC prevention in humans (reviewed by [7,8]).

The mechanisms by which EPA and DHA exert anti-cancer effects have been hypothesized to be related to their effect at the cell membrane (reviewed in [7,9,10]). It is well established that EPA and DHA are readily incorporated into membrane phospholipids (PL) [11,12,13,14,15,16] and the lipid microdomains in the membrane (lipid raft [13,17]) of BC tumour cells. This has been shown to disrupt the structural integrity of the lipid bilayer [18] and, as a result, leads to the displacement or sequestration of membrane proteins [13,19,20] involved in cell survival (reviewed in [8,21]). The epidermal growth factor receptor (EGFR) is of particular importance in BC as it is often truncated or overexpressed and is found in lipid rafts [8,22]. When DHA and EPA are incorporated into lipid rafts and membrane PL, the localization and phosphorylation of EGFR has been altered [23,24,25].

Current literature regarding BC prevention seldom considers the effects of EPA and DHA separately and refers to them collectively as n-3 LCPUFA, even though dietary sources of n-3 LCPUFA from whole foods or supplements differ dramatically in the concentrations and ratios of EPA:DHA (reviewed in [10]). The structural differences between EPA and DHA (reviewed in [26]), suggest that there may be differences in membrane incorporation and subsequent effects on membrane fluidity and function. There is evidence that these fatty acids (FA) have different biological effects in markers of cardiovascular disease risk [27] and neurological and neurodegenerative diseases (reviewed in [28]). Despite this, there are a limited number of studies that have explored the relative efficacy of DHA compared to EPA and how mixtures compare in vitro and in feeding models of BC. The objective of the present manuscript is to review the literature to determine, in BC models, the relative anti-cancer effect of EPA and DHA alone and in mixtures on tumour cell viability, apoptosis, proliferation and incorporation into cellular lipids.

## 2. Methods

### 2.1. Search Criteria

The present review took a systematic approach to analyzing the literature and included studies that met the following criteria: (1) in vitro studies that compared the effect of DHA to EPA and/or different EPA:DHA mixtures on anti-cancer outcomes in human BC cell lines, or (2) feeding studies that compared the effect of supplementing the diet with EPA, DHA, or different EPA:DHA mixtures in rats with induced mammary carcinogenesis or mice bearing human BC tumours. A literature search of in vitro and feeding studies was conducted in Medline/OVID database on 20 June 2017 and Elton B. Stephens (EBSCO) host database on 1–4 July 2017 using the following terms including: “fatty acids, omega-3, polyunsaturated fatty acids, docosahexaenoic acids, DHA, eicosapentaenoic acid, EPA, marine oil, fish oil(s)”. Keywords including “anticancer; anti-cancer; breast or mammary neoplasms; experimental; carcinoma, ductal; triple negative breast neoplasms; cell line, tumour; MCF-7 cells; SK-BR-3; MDA-MB-231; neoplasms; heterografts; triple negative or HER2 positive; Mammary Neoplasms, Experimental”/ci [Chemically Induced]; rats, transgenic or Sprague Dawley; mice, transgenic or nude or knockout or athymic” were used to capture relevant BC literature. No restriction was made on publication date. The search was rerun on 26 September 2017 in both databases to ensure relevant articles were included.

### 2.2. Characteristics of Included Studies

In total, 21 studies met the search criteria including 16 in vitro studies and 5 feeding studies. Of the included in vitro studies, 15 directly compared EPA to DHA [11,12,17,23,24,25,29,30,31,32,33,34,35,36,37], while 3 analyzed different EPA:DHA mixtures [13,29,32]. Of the three studies that used EPA:DHA mixtures, none of these compared the effect of these combination treatments to EPA and DHA alone. FA concentrations used in these studies are of physiological relevance as fish oil supplementation in non-small cell lung cancer patients resulted in plasma PL EPA levels equivalent to approximately 88 μM [38]. Of the included feeding studies, three compared the effect of feeding EPA and DHA [14,15,39], while 2 compared mixtures [16,40]. One of the studies comparing a EPA alone diet and DHA alone diet also examined a 1:1 EPA:DHA diet [39].

### 2.3. Data Extraction and Standardization

For each of the studies included, data was extracted on study design (BC subtype, BC model, treatment groups, concentration of EPA and/or DHA, exposure period, assays) and effect on anti-cancer outcome measures (plasma membrane incorporation, cell growth and viability, EGFR, apoptosis, and phosphoinositide-3-kinase/protein kinase B (PI3K/Akt) signaling). To synthesize the literature in a clear, concise, and consistent manner, data from included studies was standardized after analysis of reported tables and graphs. To standardize the method of reporting concentrations of EPA and/or DHA, concentrations were standardized to micromolar (µM) from in vitro studies and g/100 g diet (*w/w*) for feeding studies. To standardize the data related to anti-cancer outcome measures, data from in vitro and feeding studies were standardized to fold change or percent change, as appropriate.

## 3. The Incorporation of Eicosapentaenoic Acid (EPA) and Docosahexaenoic Acid (DHA) into Tumour Cell Lipids

EPA and DHA are readily incorporated into tumour lipids, [32], PL [11,12,13,17,24] and plasma membrane raft PL [13,17] of triple negative (ER−, PR−, HER2−) MDA-MB-231 [11,12,13,17,24,32] and ER+ MCF-7 BC cells [11,12,17,24,32]. An increase in EPA and/or DHA into tumour cell lipids and PL was found to decrease cell survival, as determined by decreased cell viability [11,13,29,32,33,36,37] and proliferation [12,23,24,25,30,31,32,34,35] as well as increased apoptosis [12,23,24,25,29,34,35,37].

The relative increase of EPA and DHA with EPA:DHA mixtures into the plasma membrane has been assessed in MDA-MB-231 [13,32] and MCF-7 [32] BC cells exposed to a 1.5:1 EPA:DHA ratio. The fold increase in EPA was more than DHA in whole cell PL and lipid raft PL [13,32]. If EPA and DHA were equally incorporated, it could be predicted that the amount of EPA in the membrane would be approximately 1.5 times that of DHA in a 1.5:1 EPA:DHA mixture. However, researchers found that the fold increase in EPA was at least twice that of DHA in whole cell and lipid raft PL in both BC subtypes, showing that there is preferential uptake of EPA (Table 1). The fold increase in DHA in MDA-MB-231 BC membrane lipids was reported to be higher than EPA when arachidonic acid (AA) was provided at 140 μM in the media [32]. EPA and AA compete for D5-desaturase [41] and PL uptake into the plasma membrane [42], which may explain why EPA was not preferentially taken up in the presence of a high concentration of AA. Additionally, in this study the fold increase of EPA in MCF-7 BC membrane lipids was greater than that of MDA-MB-231 BC cells with the same EPA:DHA mixtures [32].

A feeding study by Yuri et al. [39] with a 1:1 EPA:DHA mixture found the fold increase of DHA was approximately double of EPA (23 vs. 14 fold increase), which is higher than what would be predicted if equivalent uptake into the membrane occurred. However, when a n-3 LCPUFA diet with more EPA than DHA (1:0.75 EPA:DHA) was fed to rats with induced mammary carcinogenesis, the fold increase in EPA in tumour lipids was greater than what would be predicted [16]. Wei et al. [40] compared the effects of feeding five EPA:DHA diets to rats with induced mammary carcinogenesis. All diets had a 1:5.5 EPA:DHA ratio, but varied in the total concentration (*w/w*) of EPA and DHA (Table 2). When the total concentration of EPA + DHA (*w/w*) was low, the amount of EPA in tumour lipids was greater than predicted [40], whereas feeding the diet with the highest concentration of EPA + DHA (*w/w*) resulted in more DHA in tumour lipids. Collectively, these studies suggests that EPA is preferentially incorporated with EPA:DHA mixtures. It is likely that Yuri et al. [39] and Wei et al. [40] saw more DHA in the membrane due to a concentration effect, as these researchers used much higher concentrations of EPA and DHA (*w/w*) in their EPA:DHA diets than other studies included in the present review (9.5 g/100 g and 6.6 g/100 g *w/w*, respectively) (Table 2).

When comparing EPA and DHA directly at the same concentration, preferential uptake into tumour lipids or PL differs between tumour cell membrane location (whole cell lipids or lipid raft) and BC subtype (Table 3). In MDA-MB-231 BC cells, more EPA was found in whole cell lipids [24] and PL [11,12], while DHA appears to be more concentrated into lipid rafts [17]. In MCF-7 BC cells, the fold increase of EPA is similar to DHA in whole cell lipids [24] and PL [11], while the amount of EPA is greater than DHA in lipid rafts [17]. This shows that there are distinctions between BC subtypes and that measurement of whole cell PL may not be reflective of changes in lipid raft PL. In feeding studies, the amount of DHA found in tumour cell lipids is greater than that of EPA at the same concentration [14,15] (Table 4). DHA has also been shown to increase to a greater extent than EPA in tumour PL after long-term feeding (13 weeks), but this was not apparent in short-term feeding (1 week) [15], showing that the exposure period to DHA and EPA is an important consideration to determine the relative efficacy of fold increases into tumour PL. EPA and DHA are enzymatically cleaved from the plasma membrane by phospholipase A2 under inflammatory stimuli [42]. EPA’s hydrocarbon backbone is the same length as that of AA (C20:5 n-3) [42]; therefore, EPA acts as a substrate for cyclooxygenase (COX) in the eicosanoid synthesis pathway and produces prostaglandin (PGE_3_) [7,43]. DHA is a longer n-3 LCPUFA than EPA (C22:6 n-3) and cannot act as a substrate for COX, although it is able to bind and inhibit COX [7]. Therefore, it could be hypothesized that since EPA, and not DHA, is readily cleaved and used as a substrate for eicosanoid synthesis there appears to be less EPA than DHA in tumour PL.

In summary, in vitro studies with strictly controlled environmental conditions show that the fold increase of EPA in plasma membrane is greater than DHA when provided as a single n-3 LCPUFA and in EPA:DHA mixtures, providing mechanistic evidence for preferential incorporation. In feeding studies, DHA appears to increase to a greater extent into tumour lipid and PL fractions, while EPA is preferentially incorporated in mixtures. It is possible that in feeding studies when EPA is combined with DHA, EPA’s effect on membrane-mediated processes is altered.

## 4. Effect of DHA and EPA on Tumour Cell Survival

### 4.1. Cell Growth and Viability

There is considerable evidence that exposing BC cells to EPA and DHA significantly reduces survival (Table 5). In these studies, growth and viability was measured using a number of different methods, including trypan blue exclusion [11,25,29,36] colony formation assays [37], and changes in MTS [31], MTT [12,23,24,32,34,35], and WST-1 [30] measures of metabolic activity, which likely contributes to the wide range in efficacy reported. Studies have also reported decreases in the activation of the PI3K/Akt proliferative signaling pathway [23,25] and phosphorylation of EGFR [24,25]. EPA and DHA also increase proteins involved in apoptotic signaling [12,23,24,37].

Although both EPA and DHA alter viability, their incorporation into tumours is not the same and likely their mechanisms are different. Few researchers have attempted to find the ratio and concentration of EPA and DHA that optimally reduces BC cell survival (Table 5). Mansara et al. [32] and Schley et al. in 2005 and 2007 [13,29] examined the effect of EPA and DHA at a ratio of 1.5:1 (EPA:DHA). Both studies reported decreases in BC cell viability in experiments that ensured a sufficient n-6 FA supply by providing either linoleic acid (LA) [13,29] or AA [32] in the media.

The majority of studies directly comparing the relative efficacy of DHA to EPA have shown that DHA decreases cell viability to a greater extent in MDA-MB-231 [12,25,31,33], MCF-7 [11,24,29,34,35,37], SK-BR-3 [30] and 4HT1 [35] BC cells (Table 6). No studies found that DHA and EPA increase cell viability [24,32,36]. The greater anti-cancer effect of DHA occurred in most of these studies despite EPA being incorporated into tumour lipids and PL fractions to a greater extent than DHA in MDA-MB-231 BC cells [11,12,24] and similar incorporation to that of EPA in MCF-7 BC cells [11,24]. This suggests that DHA alters tumour cell survival differently than EPA and that simply measuring the relative amount that is incorporated into lipids does not explain the difference in efficacy. EPA and DHA are established precursors for anti-inflammatory lipid mediators [7,10,43]. Lipoxygenase and COX pathways use EPA as a substrate for the synthesis of E-series resolvins and DHA is used to produce D-series resolvins, protectins, and maresins [7,42]. These lipid mediators are cytoprotective in normal cells [7,44]. The role of resolvin and protectins in cancer has not been fully elucidated [45,46]. Due to their potent anti-inflammatory properties, it has been hypothesized that resolvins attenuate inflammation-related carcinogenesis [45]. Although not yet investigated in BC [47], it is possible that in E- and D-series resolvins may have distinct effects on cytotoxicity and may account for differences in cell viability.

There is conflicting evidence surrounding the relative efficacy of DHA and EPA on BC cell survival. Das et al. [36] showed that EPA decreases cell viability to a significantly greater extent than DHA after 3 days in luminal B (ER+ PR−/+ HER2+) ZR-75-1 BC cells. DHA and EPA have been shown to be equally efficacious in MDA-MB-231 BC cells using an oleic/LA FA background mixture [11]. Researchers have observed concentration dependent effects of EPA and DHA on survival in MDA-MB-231 [24,30,34], MDA-MB-435s [34], and MCF-7 BC cells [23,30]. Ewaschuk et al. [30] identified a concentration gradient in MCF-7 cells, where DHA decreased cell viability to a greater extent at lower concentrations and EPA was more efficacious at killing BC cells at higher concentrations [24,30]. However, Ewaschuk et al. [30] did not statistically examine the differences in the effects of DHA and EPA. More efficacious killing was observed when DHA was provided in low amounts, suggesting that DHA is more potent. Triple negative BC (TNBC) cells have also been reported to have concentration gradients; however, there is conflicting evidence on the relative efficacy of EPA and DHA at high and low concentrations [24,30,32,34]. The difference in the relative efficacy seen in these studies is likely due to the way EPA and DHA were delivered to the tumour cells as some deliver the n-3 LCPUFA bound to either bovine serum albumin (BSA) [11,30], delipidated endotoxin free BSA [32], or dissolved in ethanol [12,23,24,25,29,33,34,36]. FA that are dissolved in ethanol are not bound to protein and are more readily accessible for incorporation into BC cells, which may induce cytotoxic effects [48].

In summary, EPA and DHA when provided alone or in a mixture, reduce survival of triple negative, ER+, luminal B, and HER2+ BC cells in vitro, although when compared at the same dose, DHA appears to be more efficacious. This might be explained by the structural differences between DHA and EPA. DHA (C22:6 n-3) has one more double bond than EPA (C20:5 n-3) and a longer hydrocarbon chain, giving DHA a distinct three-dimensional conformation that may disrupt the highly ordered cellular membrane to a greater extent [20,26].

### 4.2. Epidermal Growth Factor Receptor (EGFR)

Of the many receptors involved in growth, the EGFR has been studied the most in n-3 LCPUFA studies. The EGFR is an important membrane receptor that regulates growth and possibly apoptosis in BC cells [13,23,24,25]. EGFR is activated by phosphorylation [22] and both EPA and DHA have been shown to alter EGFR phosphorylation in human BC cells [24]. A study conducted by Schley et al. [13] reported that EPA:DHA mixtures increased whole cell phosphorylated EGFR (pEGFR) and decreased lipid raft EGFR. There was no significant change in whole cell EGFR, implying that EPA:DHA mixtures changed the activation and translocation of EGFR but not total levels of EGFR (Table 7). Increased pEGFR is typically associated with increases in proliferation [50]; however, Schley et al. [13] also observed a decrease in cell viability and an increase in phosphorylated p38 MAPK in cells incubated with this EPA:DHA mixture, which is proposed to promote apoptosis by phosphorylating BimEL, a pro-apoptotic Bcl-2 protein [51,52].

In Corsetto et al. [24], treatment with DHA decreased whole cell EGFR and pEGFR to a greater extent than EPA in MDA-MB-231 BC cells [24]. When 0.01 μM epidermal growth factor (EGF) was added to the media with EPA or DHA treatments, EPA decreased pEGFR to a greater extent and the effect of DHA on EGFR was blunted (Table 8). Lee et al. [25] compared the effect of DHA and EPA on whole cell EGFR in MDA-MB-231 BC cells. Western Blot Analysis showed that DHA decreased amount of EGFR, while EPA did not have a visible effect. Unfortunately, these researchers did not quantify the effect on EGFR. Collectively, this data suggests that DHA is more efficacious than EPA when provided as a single FA in TNBC. In MCF-7 BC cells, treatment with either DHA or EPA did not significantly change the ratio of whole cell pEGFR:EGFR [23].

To summarize, EPA:DHA mixtures significantly increase whole cell pEGFR and decrease lipid raft EGFR in MDA-MB-231 BC cells. DHA decreases whole cell EGFR and pEGFR to a greater extent than EPA when provided as a single FA in these BC cells, suggesting that the effects of EPA:DHA mixtures are attributable to the presence of DHA. The EGFR typically partitions into the lipid raft; however, changes in the lipid bilayer are associated with decreases in EGFR [53]. Since MDA-MB-231 BC cells favour incorporation of DHA into the lipid raft, it is plausible that DHA disrupts the structural integrity of the lipid raft and affects EGFR localization and phosphorylation status. In ER+ MCF-7 BC cells, DHA and EPA act differently and there is not an effect in ER+ cells (MCF-7) on EGFR. This could be attributed to the preferential uptake of EPA into lipid rafts compared to DHA [17], which does not have the same spatial conformation as DHA [26] and, as a result, may not affect receptors found in the lipid raft.

## 5. Effect of DHA and EPA on Tumour Cell Death

### 5.1. Apoptosis

EPA and/or DHA have pro-apoptotic effects in both triple negative [12,24] and ER+ [23,24,37] BC subtypes. The pro-apoptotic effects occur with increases in plasma membrane incorporation [12,24] and decreases in cell viability [12,23,24,37], PI3K/Akt activation, [23], and pEGFR activation [24]. These data suggest that EPA and DHA may affect multiple steps in apoptosis.

Mixtures of EPA and DHA with or without LA have been shown to significantly increase apoptosis of MDA-MB-231 BC cells as indicated by an increase in activated caspases [29] (Table 9). The presence of LA in the media blunted the observed increase in activated caspases as well as decreases in cell viability and Akt phosphorylation, suggesting that the efficacy of EPA:DHA combination treatments is dependent upon the presence of other FA.

In TNBC cell lines, DHA caused greater decreases in total amounts of Bcl-2 and procaspase 8 [24] as well as larger increases in single stranded DNA when compared to EPA [12] (Table 10). These data suggest that DHA is a more efficacious inducer of apoptosis, which may be related to the preferential incorporation of DHA into lipid rafts and its more potent effect on decreasing cell viability and whole cell EGFR [25] and pEGFR [24].

In Corsetto et al. [24], researchers examined changes in Bcl-2 and procaspase 8 in ER+ MCF-7 BC cells. DHA had more of an effect on decreasing procaspase 8 and EPA had a larger effect on decreasing Bcl-2. Of note, these endpoints are not valid markers of apoptosis in MCF-7 BC cells, which do not express caspase-3 [54], a critical effector caspase in the apoptosis signaling cascade [55]. These BC cells rely on caspases 6, 7, and 9 to initiate apoptosis [54]; therefore, procaspase 8 is not a central part in the initiation of MCF-7 BC cells. In addition, MCF-7 BC cells are associated with increased total Bcl-2; therefore, it may be easier to see differences than with a cell line that does not overexpress this protein [56]. Chamras et al. [37] found significant increases in the number of apoptotic cells with EPA and DHA treatments when provided alone in MCF-7 BC cells; however, these increases were not different between treatment groups.

In summary, the published data suggests that DHA and EPA have similar effects on apoptotic signaling in ER+ BC cells. This phenomenon may also be explained by previous work conducted in BC cells with DHA, and cell death receptors. In Ewaschuk et al. [30], treatment of MDA-MB-231 BC cells with DHA caused the CD95 death receptor to be translocated to lipid rafts for apoptosis induction. This demonstrates that DHA regulates membrane-associated proteins associated with extrinsic apoptosis. The reliance of MCF-7 BC cells on the intrinsic pathway through the initiation of capsase-9 suggest that the effect of DHA on the membrane and subsequent effects on cell death membrane receptors in TNBC would not impact apoptosis to the same extent in ER+ BC.

Researchers have yet to compare and contrast the effect of DHA and EPA on autophagy, a conserved process that involves the sequestration and degradation of cellular components [57]. Jing et al. [57] reported that in MCF-7 BC cells, DHA induces AMPK phosphorylation, and a decrease in p53 expression and mTOR signaling. mTOR is a negative regulator of autophagy; therefore, DHA promotes autophagy, decreases cell viability, and increases tumour cell susceptibility to apoptosis [57].

A decrease in pAkt is reported upon incubation of MCF-7 BC cells with DHA (see Section 5.2). When Akt is activated, it removes the inhibition of TSC1/2 on Rheb, facilitating the activation of the mTORC1 complex (Raptor and mTOR), which promotes protein synthesis and cell growth [58]. It is plausible that the observed decrease in Akt activation with DHA is a consequence of inhibition of the activation of mTOR in the mTORC1 complex through p53 (as seen in Jing et al. [57]). Increases in apoptosis and decreases in cell viability were also found in MCF-7 BC cells, consistent with the induction of autophagy and promotion of apoptosis as reported by Jing et al. [57].

### 5.2. The Phosphoinositide-3-Kinase/Protein Kinase B (PI3K/Akt) Pathway

The PI3K/Akt signaling pathway is a proliferative signaling pathway that has been implicated in BC pathogenesis [59]. EPA and DHA have been investigated for their ability to regulate the phosphorylation and activation of Akt, a serine/threonine kinase that regulates cell survival, growth, and transcription [60,61]. One study by Schley et al. [29] examined the effect of a EPA:DHA mixture on the PI3K/Akt pathway (Table 11). Researchers compared the effects of EPA and DHA with or without LA in the media on phosphorylated Akt (pAkt) and Akt in MDA-MB-231 BC cells. They observed a 47% decrease of pAkt; however, this decrease was blunted by the presence of LA. This demonstrates that the presence of n-6 FA may decrease the effect of EPA and DHA on Akt phosphorylation. No significant changes in Akt were observed, suggesting that the combination of DHA and EPA decreases phosphorylation but not total Akt.

Lee et al. [25] saw a decrease in total Akt with DHA and not EPA in MDA-MB-231 BC cells. This may be due to higher lipid raft PL incorporation of DHA [17], a greater decrease in cell viability [12,25,29,31,33], higher apoptosis [12,24], and lower levels of pEGFR [24] and EGFR [25] in tumour cells with DHA treatment compared to MDA-MB-231 BC cells treated with EPA. It is likely that higher lipid raft PL incorporation of DHA decreased EGFR and resulted in a decreased activation of the downstream PI3K/Akt pathway. This may have blunted or removed Akt’s inhibitory effect on Bad and the intrinsic apoptotic signaling cascade [55].

Cao et al. [23] found that EPA and DHA decrease the ratio of pAkt:Akt in MCF-7 BC cells to the same extent, albeit statistical analysis was not performed in this study (Table 12). Western blot analysis showed that EPA and DHA decrease the pAkt:Akt ratio by decreasing pAkt and had no effect on total Akt [23]. This may be explained by the lack of effect of EPA and DHA on total EGFR in these BC cells [23]. PI3K and Akt are kinases that are subsequently activated in a signaling cascade upon binding of EGF to EGFR, receptor dimerization, and EGFR activation by phosphorylation [62]. In controlled in vitro conditions, if EGFR phosphorylation status does not change with exposure to EPA and/or DHA, neither will Akt unless activated by another stimuli.

The effect of feeding EPA and DHA on Akt phosphorylation status has been examined by Chen et al. [63]. These authors intragastrically delivered either a low EPA:DHA diet (0.42 g/100 g diet EPA and 0.38 g/100 g diet DHA) or a high EPA:DHA diet (3.12 g/100 g diet EPA and 1.58 g/100 g diet DHA) to Sprague Dawley Rats bearing N-Nitroso-N-methylurea (MNU) induced mammary carcinogenesis. Rats provided the high EPA:DHA diet experienced a decrease in tumour size and multiplicity compared to the low EPA:DHA group, albeit no statistical analysis was conducted [63]. Western blot analysis revealed no significant differences in Akt between groups; however, a significantly lower level of pAkt (S473) was observed in the tumours of rats fed the high EPA:DHA diet [63]. Researchers did not find a significant difference between groups on the phosphorylation status of the T308 residue of Akt [63]. Phosphorylation of both S473 and T308 are required for full Akt activation [64]; therefore, the decrease in S473 observed with the high n-3 diet most likely affected the function of pAkt, but did not inhibit pAkt activation. The results of this study demonstrating that feeding DHA and EPA can decrease Akt activation in tumours is consistent with the results of the in vitro BC cell studies. This study also shows that western blot analysis of membrane receptors and phosphorylation status of a single residue may not directly translate to protein activity; therefore, functional assays should also be considered.

## 6. Summary, Conclusions and Future Directions

EPA and DHA have demonstrated anti-cancer effects across a variety of cancer types (reviewed in [7]). Currently, it is not known if EPA and DHA have similar effects on BC tumours and if mixtures alter their effect on tumour cell viability, apoptosis, proliferation and incorporation into cellular lipids. Research conducted in BC cell lines and animal models provide essential evidence for changing BC treatments. However, the findings from these studies need to be validated in clinical trials before they can be used to change recommendations or treatment of BC patients.

**Figure 1:**
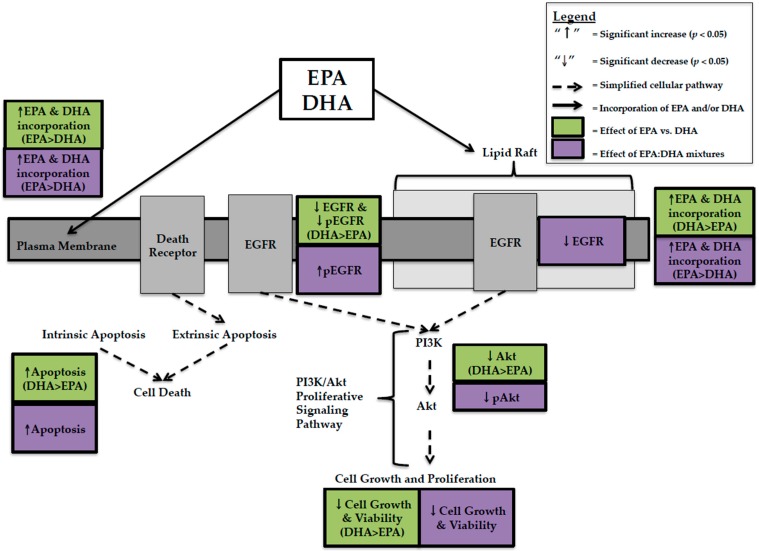
Schematic illustrating the pleiotropic effects and relative efficacy of eicosapentaenoic Acid (EPA) and/or docosahexaenoic Acid (DHA) in MDA-MB-231 human breast cancer cells. EGFR = Epidermal Growth Factor Receptor; pEGFR = Phosphorylated EGFR; PI3K = Phosphoinositide-3-kinase; Akt = Protein Kinase B; pAkt = Phosphorylated Akt.

In the present review, EPA and DHA were compared at the same concentration, DHA had higher anti-cancer activity in TNBC cells. This was explained by greater decreases in cell viability [12,25,29,31,33], EGFR [24,25], pEGFR [24], Akt [25], and greater increases in apoptosis [12,24] with DHA compared to EPA (Figure 1). This effect was not predicted by whole cell lipid incorporation as EPA was incorporated more [24], but is related to lipid raft incorporation where DHA was preferentially incorporated over EPA [17]. The data suggests that DHA’s spatial conformation disrupts the organization and fluidity of the lipid raft bilayer in BC cells, effecting membrane receptors involved in proliferative signaling pathways. In MCF-7 BC cells, DHA caused greater decreases in cell viability than EPA when provided at the same concentration. However, unlike TNBC this could not be explained by changes in EGFR, pEGFR [23], Akt [23], or apoptosis [23]. We hypothesized that this was due to preferential incorporation of EPA into lipid rafts of MCF-7 BC cells [17]. EPA has a smaller, more rigid spatial conformation [26] and, as a result, may not affect receptors and proteins involved in proliferative and apoptotic signaling to the same extent as DHA but may affect other proteins. The production of distinct lipid mediators from EPA and DHA (E- and D-series resolvins) may also account for differences in cytotoxicity; however, this remains to be tested in human BC cells representing distinct BC subtypes.

Few studies have attempted to compare and contrast the effect of feeding EPA and DHA alone [14,15,39]. In contrast to in vitro studies, feeding diets supplemented with DHA, compared to EPA resulted in higher membrane incorporation of DHA [14,15,39]. This discrepancy between preferential incorporation of EPA in vitro and DHA in feeding studies may be due either to an interaction between EPA and DHA at the level of the membrane, decreasing availability for membrane uptake or it could be due to inherent differences between cells in vitro and tumour models in animals. In vitro studies allow for the strict control of experimental conditions as well as the precise and accurate delivery of EPA and/or DHA. The presence of the gastrointestinal and hepatic portal vein systems in feeding trials effects how n-3 LCPUFA are absorbed and distributed, therefore; the concentrations of EPA and DHA presented to the tumour in feeding studies may differ compared to what is in the experimental diet.

EPA:DHA mixtures have been studied at a ratio of 1.5:1 and decrease cell viability in both MDA-MB-231 and MCF-7 BC cells [13,32]. There was more EPA in vitro than predicted by this 1.5:1 ratio into whole cell lipids, PL, and lipid raft PL [13,32], again demonstrating that the ratio of EPA:DHA provided in the diet does not predict membrane incorporation. EPA:DHA mixtures in feeding studies [16,39,40] also did not predict membrane incorporation. Despite this, in MDA-MB-231 BC cells, 1.5:1 EPA:DHA mixtures decreased proliferative signaling by decreasing Akt activation [29] and increased apoptosis through caspase activation [29] and phosphorylation of whole cell EGFR [13]. In vitro studies [13,32] have not compared mixtures to EPA and DHA alone on proliferative and apoptotic signaling pathways, making it difficult to determine if the anti-cancer effect of mixtures can be predicted on these endpoints. However, a feeding study conducted by Yuri et al. [39] found that a 1:1 EPA:DHA diet and EPA alone decreased tumour multiplicity (number of carcinomas per effective rat) to a similar extent (1.59 and 1.67, respectively) but to a lesser extent than DHA alone (0.23). This study also found that DHA was preferentially incorporated into mammary tissue lipids to a greater extent than predicted, confirming that the dietary ratio does not predict the relative amount of DHA in the membrane or changes in tumour cell proliferation [39].

Presently there is a great deal of heterogeneity in the literature that makes it challenging for researchers to directly compare and contrast findings from various studies. The exposure period, ratios, and concentrations of EPA and/or DHA used in vitro and in feeding studies differ between studies. Few in vitro studies include a control FA condition [11,13,29] or use background FA that are of physiological relevance [11,13,29,32]. Similarly, few feeding studies used a basal diet to compare the effects of EPA and/or DHA diets [14,15]. In addition, a wide range of assays each with their unique set of strengths and limitations are used across studies to determine the effect of EPA and/or DHA on BC cell viability (Table 6) and apoptosis (Table 10).

Oftentimes, feeding studies did not record food intake [15,16,39], which is critical to determine if animals consumed a sufficient amount of the experimental diet to be exposed to the intended concentration of EPA and DHA. In the present review, feeding studies either induced mammary carcinogenesis by administering MNU [39,40,63] or implanted MDA-MB-435 human BC cell lines in the mammary fat pad of rodents [14,15,16]. Both of these models have inherent limitations. The carcinogenicity of MNU can vary based on the route of administration, timing of exposure, and dose [65], which varied between studies. Xenograft models that inject human BC cells into the mammary tissue more closely represent the tumour microenvironment and BC tumour progression; however, the MDA-MB-435 cells used by Rose et al. [14,15,16] originated from a melanoma cell line and are spontaneously metastatic [6] and, as a result, may not accurately represent BC pathogenesis.

Several knowledge gaps exist in the current literature that need to be addressed before the pleiotropic effects and relative efficacy of EPA and DHA in BC subtypes are fully characterized. In the present review, studies that investigated the anti-cancer effects of EPA, DHA, or EPA:DHA mixtures in vitro were primarily studied in MDA-MB-231 and MCF-7 BC cells. Other BC subtypes including HER2+ and luminal B BC have not been studied. There is also a lack of feeding studies that verify the mechanistic data from in vitro studies. The present review showed that preferential incorporation of EPA and DHA differed between in vitro and some feeding models. Further, membrane changes (fluidity) maybe important in driving the mechanism(s) of cellular phenotypes (apoptosis, proliferation etc.). It would be beneficial to further examine EPA, DHA, and EPA:DHA mixtures in well-designed pre-clinical models. This could include either: (1) in vitro models that mimic the in vivo tumour microenvironment (such as three-dimensional cell culture; reviewed in [66,67]) or (2) animal models that represent the heterogeneity of human tumours (such as patient derived xenografts implanted into mammary tissue; reviewed in [68,69]). HER2 is another ErbB receptor that is commonly truncated or overexpressed in BC [22,70]. Evidence exists for a beneficial effect of DHA [25,71,72] on HER2. However, to date, there are no studies that have systematically compared and contrasted the effect of EPA, DHA, and EPA:DHA mixtures in HER2 overexpressing human BC cell lines, warranting further research. Lastly, the effect of EPA and DHA on autophagy should be examined in MCF-7 BC cells as DHA has been shown to promote autophagy and apoptosis through p53 in these cells [57]. Research could also be done to identify if there is an effect of EPA and/or DHA on autophagy in MDA-MB-231 BC cells, a cell line with a mutated *p53* gene [73].

## Figures and Tables

**Table 1:** Incorporation of EPA and DHA measured in the plasma membrane with EPA:DHA mixtures in human BC cell lines.

Citation	Cell Line	Concentration of EPA or DHA (µM)	Ratio EPA:DHA	Lipid Fraction	Fold Change in Incorporation *	
					EPA	DHA
Schley, Brindley and Field [13]	MDA-MB-231	60 EPA + 40 DHA	1.5:1	Whole cell PL	↑157	↓0.2
				Lipid raft PL	↑73	↑8
		45 EPA + 30 DHA + 75 LA	1.5:1	Whole cell PL	↑49	↑2
				Lipid raft PL	↑21	↑3
Mansara, Deshpande, Vaidya and Kaul-Ghanekar [32]	MDA-MB-231	84 EPA + 56 DHA + 140 AA	1.5:1	Whole cell total lipids	↑0	↑1
		120 EPA + 80 DHA + 80 AA			↑1	↑0.2
		134 EPA + 90 DHA + 56 AA			↑1	↑1
		140 EPA + 93 DHA + 47 AA			↑2	↑1
		153 EPA + 102 DHA + 25 AA			↑4	↑1
	MCF-7	84 EPA + 56 DHA + 140 AA	1.5:1	Whole cell total lipids	↑1	↑0.3
		120 EPA + 80 DHA + 80 AA			↑2	↑1
		134 EPA + 90 DHA + 56 AA			↑3	↑1
		140 EPA + 93 DHA + 47 AA			↑3	↑1
		153 EPA + 102 DHA + 25 AA			↑5	↑1

EPA = eicosapentaenoic acid, DHA = docosahexaenoic acid; LA = linoleic acid; AA = arachidonic acid; PL = phospholipid; FA = fatty acids; “↑” denotes significant increase (*p* < 0.05); “↓” denotes significant decrease (*p* < 0.05). * Fold change relative to control conditions.

**Table 2:** Incorporation of EPA and DHA measured in the plasma membrane when feeding EPA:DHA mixtures in rodent models of human BC.

Citation	Method Used to Induce Mammary Carcinogenesis	Experimental Diets	Concentration of EPA or DHA (*w/w* Diet, g/100 g)	Ratio EPA:DHA	Lipid Fraction	Exposure (Weeks)	Fold Change in Incorporation *	
							EPA	DHA
Yuri, Danbara, Tsujita-Kyutoku, Fukunaga, Takada, Inoue, Hada and Tsubura [39]	MNU administration in rats	EPA	9.5 EPA	1:0	Mammary tissue total lipids	20	↑*31*	↑*0.5*
		DHA	9.5 DHA	0:1			↑ *2*	↑*30*
		EPA + DHA	4.75 EPA + 4.75 DHA	1:1			↑*14*	↑*23*
Wei, Wang, Zhang, Mi, Zhu, Yu, Yuan, Chen, Wang and Chang [40]	MNU administration in rats	SFA	0 EPA + 0 DHA	0:0	Tumour total lipids	18	ND	↑0.04
		MUFA	0 EPA + 0 DHA	0:0			ND	↑0.5
		n-6 PUFA	0 EPA + 0 DHA	0:0			ND	↓0.2
		n-3 LCPUFA	1 EPA + 5.6 DHA	1:5.5			↓0.3	↑0.3
		1:1 (n-6:n-3)	0.5 EPA + 2.8 DHA				↑0.04	↓0.07
		1:2:1 S/M/P 1:1 (n-6:n-3)	0.2 EPA + 1.1 DHA				↑0.1	↑0.04
		5:1 (n-6:n-3)	0.16 EPA + 0.9 DHA				↑0.5	↓0.05
		10:1 (n-6:n-3)	0.09 EPA + 0.49 DHA				↑0.07	↓0.06
Rose, Rayburn, Hatala and Connolly [16]	Xenograft in mammary fat pad using MDA-MB-435 in nude mice	11.5% MO+ 11.5% CO	0.42 EPA + 0.32 DHA	1:0.75	Tumour PL	12	↑1	↑0.2
		18% MO+ 5% CO	0.66 EPA + 0.50 DHA				↑3	↑0.4

Italicized numbers represents fatty acid composition of the mammary tissue and not fold-increase in incorporation, as this study did not have a control group. MNU = *N*-methyl-*N*-nitrosourea; EPA = eicosapentaenoic acid, DHA = docosahexaenoic acid; SFA = saturated fatty acid; MUFA = monounsaturated fatty acid; PUFA = polyunsaturated fatty acid; S/M/P = saturated/monounsaturated/polyunsaturated; MO = menhaden oil; CO = corn oil; PL = phospholipid; “↑” denotes significant increase (*p* < 0.05); ND = not determined; “↓” denotes significant decrease (*p* < 0.05). * Fold change relative to control conditions.

**Table 3:** Incorporation of EPA and DHA measured in the plasma membrane when comparing EPA to DHA in human BC cell lines.

Citation	Cell Line	Concentration of EPA or DHA (µM)	Lipid Fraction	Fold Change in Incorporation *	
				EPA	DHA
Corsetto, Montorfano, Zava, Jovenitti, Cremona, Berra and Rizzo [24]	MDA-MB-231	230 EPA	Whole cell total lipids	↑15	↓0.2
		200 DHA	Whole cell total lipids	↓0.8	↑7
	MCF-7	230 EPA	Whole cell total lipids	↑10	↑0.5
		200 DHA	Whole cell total lipids	↓0.6	↑9
Corsetto, Cremona, Montorfano, Jovenitti, Orsini, Arosio and Rizzo [17]	MDA-MB-231	230 EPA	Lipid raft PL	↑7	↑1
		200 DHA	Lipid raft PL	↑0.6	↑11
	MCF-7	230 EPA	Lipid raft PL	↑16	↑6
		200 DHA	Lipid raft PL	↓0.3	↑6
Yu [11]	MDA-MB-231	150 EPA + 40 OA + 40 LA	Whole cell PL	↑31	↑1.5
		150 DHA + 40 OA + 40 LA	Whole cell PL	↓0.5	↑11
	MCF-7	150 EPA + 40 OA + 40 LA	Whole cell PL	↑10	↑0.1
		150 DHA + 40 OA + 40 LA	Whole cell PL	↓0.5	↑7
Barascu, Besson, Le Floch, Bougnoux and Jourdan [12]	MDA-MB-231	30 EPA	Whole cell PL	↑13	↑2
		30 DHA	Whole cell PL	↑0.2	↑3

EPA = eicosapentaenoic acid, DHA = docosahexaenoic acid; OA = oleic acid; LA = linoleic acid; FA = fatty acids; PL = phospholipid; “↑” denotes significant increase (*p* < 0.05); “↓” denotes significant decrease (*p* < 0.05). * Fold change relative to control conditions.

**Table 4:** Incorporation of EPA and DHA measured in the plasma membrane when comparing EPA to DHA in rodent models of human BC.

Citation	Method Used to Induce Mammary Carcinogenesis	Experimental Diets	Concentration of EPA or DHA (*w/w* Diet, g/100 g)	Lipid Fraction	Exposure Period (Weeks)	Fold Change in Incorporation *	
						EPA	DHA
Rose, Connolly, Rayburn and Coleman [14]	Xenograft in mammary fat pad using MDA-MB-435 in nude mice	4% EPA	0.7 EPA	Tumour PL	13	↑54	↑26
		4% DHA	0.7 DHA			↑15	↑107
		8% EPA	1.5 EPA			↑104	↑18
		8% DHA	1.5 DHA			↑36	↑127
Rose, Connolly and Coleman [15]	Xenograft in mammary fat pad using MDA-MB-435 in nude mice	4% EPA	0.7 EPA	Tumour PL	1	↑3	↑1
		4% DHA	0.7 DHA			↑1	↑5
		8% EPA	1.5 EPA			↑5	↑1
		8% DHA	1.5 DHA			↑1	↑5

EPA = eicosapentaenoic acid, DHA = docosahexaenoic acid; PL = phospholipid; “↑” denotes significant increase (*p* < 0.05). * Fold change relative to control conditions.

**Table 5:** Comparison of EPA:DHA mixtures on cell viability in human BC cell lines.

Citation	Cell Line	Concentration of EPA or DHA (µM)	Ratio EPA:DHA	Assay	Change in Cell Viability *	Exposure (Hours)	Form of n-3 LCPUFA
Schley, Jijon, Robinson and Field [29]	MDA-MB-231	60 EPA + 40 DHA	1.5:1	TBE	↓40%	72	Dissolved in ethanol
		45 EPA + 30 DHA + 75 LA			↓31%		
Schley, Brindley and Field [13]	MDA-MB-231	60 EPA + 40 DHA	1.5:1	TBE	↓62%	72	Dissolved in ethanol
		45 EPA + 30 DHA + 75 LA			↓48%		
Mansara, Deshpande, Vaidya and Kaul-Ghanekar [32]	MDA-MB-231	84–153 EPA + 56–102 DHA + 25–140 AA	1.5:1	TBE	↓54%–↓82%	24	Conjugated to delipidated, endotoxin free BSA
				MTT	↓15%–↓30%		
	MCF-7	84–153 EPA + 56–102 DHA + 25–140 AA	1.5:1	TBE	↓38%–↓81%	24	Conjugated to delipidated, endotoxin free BSA
				MTT	↓20%–↓30%		

EPA = eicosapentaenoic acid, DHA = docosahexaenoic acid; AA = arachidonic acid; TBE = Trypan Blue Exclusion; MTT = 3-(4,5-dimethylthiazol-2-yl)-2,5-diphenyltetrazolium bromide; “↓” denotes significant decrease (*p* < 0.05); “%” = percent change from control condition; BSA = bovine serum albumin.; * Relative to control conditions.

**Table 6:** Comparison of EPA and DHA on cell growth & viability in human BC cell lines.

Citation	Cell Line	Concentration of EPA or DHA (µM)	Assay	Change in Cell Viability *	Exposure (Hours)	Form of n-3 LCPUFA	Conclusion on Relative Efficacy
Schley, Jijon, Robinson and Field [29]	MDA-MB-231	100 EPA	TBE	↓42%	72	Dissolved in ethanol	DHA > EPA
		75 EPA + 75 LA		↓30%			
		100 DHA		↓65%			
		75 DHA + 75 LA		↓58%			
Lee, Yun, Koo, Sung, Shim, Ye, Hong and Kim [25]	MDA-MB-231	5, 10, 30, & 50 EPA	MTS	↓15%–↓20%	24	Dissolved in ethanol	DHA > EPA
		5, 10, 30, & 50 DHA		↓20%–↓45%			
Corsetto, Montorfano, Zava, Jovenitti, Cremona, Berra and Rizzo [24]	MDA-MB-231	50–300 EPA	MTT	NS Δ–↓88%	72	Dissolved in ethanol	DHA > EPA (200–260 μM) & EPA > DHA (>260 μM)
		50–300 DHA		NS Δ–↓75%			
	MCF-7	50–300 EPA	MTT	NS Δ–↓75%	72	Dissolved in ethanol	DHA > EPA
		50–300 DHA		NS Δ–↓75%			
Cao, Ma, Rasenick, Yeh and Yu [23]	MCF-7	30, 60, 90, 140 EPA	MTT	↓2%–↓45%	72	Dissolved in ethanol	DHA = EPA with exception of DHA > EPA (at 90 μM) ●
		30, 60, 90, 140 DHA		↓2%–↓45%			
Ewaschuk, Newell and Field [30]	MDA-MB-231	50, 100 EPA	WST-1	↓5%–↓100%	72	Conjugated to BSA	DHA > EPA (<95 μM) & EPA > DHA (>95 μM) ●
		50, 100, 150, 200 DHA		↓45%–↓90%			
	MCF-7	50, 100 EPA	WST-1	↓25%–↓100%	72	Conjugated to BSA	DHA > EPA (<95 μM) & EPA > DHA (>95 μM) ●
		50, 100, 150 DHA		↓40%–↓100%			
	SK-BR-3	50, 100, 150 EPA	WST-1	↓5%–100%	72	Conjugated to BSA	DHA > EPA ●
		50, 100 DHA		↓80%–↓100%			
Rahman, Veigas, Williams and Fernandes [31]	MDA-MB-231	50, 100 EPA	MTS	NS Δ–↓58%	48	No information given	DHA > EPA
		50, 100 DHA		↓26%–↓74%			
Mansara, Deshpande, Vaidya and Kaul-Ghanekar [32]	MDA-MB-231	40–320 EPA	MTT	NS Δ	24	Conjugated to delipidated, endotoxin free BSA	DHA = EPA (<280 μM) & DHA > EPA (≥280 μM)
		40–320 DHA		NS Δ–↓25%			
	MCF-7	40–320 EPA	MTT	NS Δ–↓20%	24	Conjugated to delipidated, endotoxin free BSA	DHA = EPA (<200 μM), DHA > EPA (≥200 μM)
		40–320 DHA		NS Δ–↓22%			
Rose and Connolly [33]	MDA-MB-231	1.7–8.3 EPA	[^3^H] Inc.	NS Δ–↓29%	144	Dissolved in ethanol	DHA > EPA
		1.5–7.6 DHA		NS Δ–↓64%			
Barascu, Besson, Le Floch, Bougnoux and Jourdan [12]	MDA-MB-231	10–100 EPA	MTT	NS Δ–↓75%	96	Dissolved in ethanol	DHA > EPA
		10–100 DHA		NS Δ–↓85%			
Kang, Wang, Yamabe, Fukui, Jay and Zhu [34]	MDA-MB-231	12.5–200 EPA	MTT	↓0%–↓17%	72	Dissolved in ethanol	DHA = EPA (<50 μM) & DHA > EPA (>50 μM) ●
		12.5–200 DHA		↓0%–↓87%			
	MCF-7	6.25–200 EPA	MTT	↑5%–↓95%	72	Dissolved in ethanol	DHA > EPA ●
		6.25–200 DHA		↓5%–↓95%			
	MDA-MB-435s	12.5–200 EPA	MTT	↓0%–↓50%	72	Dissolved in ethanol	DHA = EPA (<50 μM) & DHA > EPA (>50 μM) ●
		12.5–200 DHA		↓0%–↓87%			
Xue, Wang, Zhao, Dong, Ge, Hou, Liu and Zheng [35]	MCF-7	25, 50, 100 EPA	MTT	↓15%, ↓25%, ↓40%	72	No information given	DHA > EPA
		25, 50, 100 DHA		↓20%, ↓33%, ↓48%			
	4T1	25, 50, 100 EPA	MTT	↓20%, ↓35%, ↓55%	72	No information given	DHA > EPA
		25, 50, 100 DHA		↓25%, ↓45%, ↓83%			
Yu [11]	MDA-MB-231	150 EPA + 40 OA + 40 LA	TBE	↓40%	48	Conjugated to BSA	DHA = EPA
		150 DHA + 40 OA + 40 LA		↓50%			
	MCF-7	150 EPA + 40 OA + 40 LA	TBE	↓45%	48	Conjugated to BSA	DHA > EPA
		150 DHA + 40 OA + 40 LA		↓58%			
Das [36]	ZR-75-1	66 EPA	TBE	↓10%	72	Dissolved in ethanol	EPA > DHA
		61 DHA		NS Δ			
Chamras, Ardashian, Heber and Glaspy [37]	MCF-7	100 EPA	Cell count	↓30%	120	No information given	DHA > EPA ●
		100 DHA		↓50%			
	MCF-7	1, 10, 100 EPA	Colony Formation	↓18%, ↓35%, ↓75%	2 weeks	No information given	DHA > EPA
		1, 10, 100 DHA		↓30%, ↓38%, ↓82%			
Yun, et al. [49]	MDA-MB-231	1–50 EPA	MTT	NS–↓55%	24	No information given	DHA > EPA
		1–50 DHA		NS–↓80%			
	MDA-MB-231	25 EPA	MTT	↓25%	36	No information given	DHA > EPA ●
		25 DHA		↓60%			
	T47D	1–50 EPA	MTT	NS–↓20%	24	No information given	DHA > EPA
		1–50 DHA		NS–↓30%			

EPA = eicosapentaenoic acid; DHA = docosahexaenoic acid; OA = oleic acid; LA = linoleic acid; TBE = Trypan Blue Exclusion; MTT = 3-(4,5-dimethylthiazol-2-yl)-2,5-diphenyltetrazolium bromide; MTS = (3-(4,5-dimethylthiazol-2-yl)-5-(3-carboxymethoxyphenyl)-2-(4-sulfophenyl)-2*H*-tetrazolium); [^3^H] Inc. = Thymidine Incorporation; WST-1 = Water-soluble Tetrazolium salt; PL = phospholipid; “↑” denotes significant increase (*p* < 0.05); “↓” denotes significant decrease (*p* < 0.05); NS Δ = no significant change; “●” = statistical significance was not assessed; “%” = percent change from control condition; BSA = bovine serum albumin; * Relative to control conditions.

**Table 7:** Change in total amounts of EGFR and pEGFR in whole cell lipids and lipid rafts with EPA:DHA mixtures in human BC cell lines.

Citation	Cell Line	Concentration of EPA or DHA (µM)	Exposure (Hours)	Change in EGFR *
Schley, Brindley and Field [13]	MDA-MB-231	60 EPA + 40 DHA	72	NS Δ in whole cell EGFR ** ↑50% whole cell pEGFR ↓lipid raft EGFR **
		45 EPA + 30 DHA + 75 LA		NS Δ in whole cell EGFR ** ↑21% whole cell pEGFR ↓lipid raft EGFR **

EPA = eicosapentaenoic acid; DHA = docosahexaenoic acid; LA = linoleic acid; EGFR = epidermal growth factor receptor; pEGFR = phosphorylated EGFR; NS Δ = no significant change; “↑” denotes significant increase (*p* < 0.05); “↓” denotes significant decrease (*p* < 0.05); “%” = percent change from control condition; * Relative to control conditions; ** Researchers did not quantify percent change in bands from control condition in Western Blot Analysis.

**Table 8:** Comparison of DHA and EPA on total amounts of EGFR and pEGFR in whole cell lipids and lipid rafts in human BC cell lines.

Citation	Cell Line	Concentration of EPA or DHA (µM)	Exposure (Hours)	Change in EGFR *
Cao, Ma, Rasenick, Yeh and Yu [23]	MCF-7	90 EPA	24	NS Δ in whole cell pEGFR:EGFR
		90 DHA		NS Δ in whole cell pEGFR:EGFR
Corsetto, Montorfano, Zava, Jovenitti, Cremona, Berra and Rizzo [24]	MDA-MB-231	230 EPA	72	NS Δ in whole cell EGFR; ↓10% whole cell pEGFR
		230 EPA + 0.01 EGF		NS Δ in whole cell EGFR; ↓52% whole cell pEGFR
		200 DHA		↓20% whole cell EGFR; ↓100% whole cell pEGFR
		200 DHA + 0.01 EGF		NS Δ in whole cell EGFR; ↓100% whole cell pEGFR
Lee, Yun, Koo, Sung, Shim, Ye, Hong and Kim [25]	MDA-MB-231	30, 50 EPA	24	NS Δ in whole cell EGFR ●
		30, 50 DHA		↓whole cell EGFR ●

EPA = eicosapentaenoic acid, DHA = docosahexaenoic acid; EGF = epidermal growth factor; EGFR = epidermal growth factor receptor; pEGFR = phosphorylated EGFR; NS Δ = no significant change; “↓” denotes significant decrease (*p* < 0.05); “●” = statistical significance was not assessed; “%” = percent change from control condition; * Relative to control conditions.

**Table 9:** Change in apoptotic markers with EPA:DHA mixtures in human BC cells.

Citation	Cell Line	Concentration of EPA or DHA (µM)	Assay	Exposure (Hours)	Effect on Markers of Apoptosis *
Schley, Jijon, Robinson and Field [29]	MDA-MB-231	60 EPA + 40 DHA	Caspase Detection Kit	72	↑29% activated caspases
		45 EPA + 30 DHA + 75 LA			↑22% activated caspases

EPA = eicosapentaenoic acid, DHA = docosahexaenoic acid; LA = linoleic acid; “↑” denotes significant increase (*p* < 0.05); “%” = percent change from control condition; * Relative to control condition.

**Table 10:** Comparison of EPA and DHA on changes in apoptotic markers in human BC cells.

Citation	Cell Line	Concentration of EPA or DHA (µM)	Assay	Exposure (Hours)	Effect on Markers of Apoptosis *
Cao, Ma, Rasenick, Yeh and Yu [23]	MCF-7	90 EPA	Flow Cytometry (Annexin V/PI)	12	↑11% apoptotic cells ●
			TUNEL		↑11% TUNEL positive cells ●
		90 DHA	Flow Cytometry (Annexin V/PI)	12	↑10% apoptotic cells ●
			TUNEL		↑9% TUNEL positive cells ●
Corsetto, Montorfano, Zava, Jovenitti, Cremona, Berra and Rizzo [24]	MDA-MB-231	230 EPA	Western Blot	72	NS Δ in Bcl-2; NS Δ in procaspase 8
		200 DHA			↓100% Bcl-2; ↓45% procaspase 8
	MCF-7	230 EPA	Western Blot	72	↓100% Bcl-2; ↓20% procaspase 8
		200 DHA			NS Δ in Bcl-2; ↓35% procaspase 8
Barascu, Besson, Le Floch, Bougnoux and Jourdan [12]	MDA-MB-231	10, 30, 50 EPA	Flow Cytometry (ssDNA)	24	↑0.6%; ↑39%; ↑79%
		10, 30, 50 DHA			↑27%; ↑63%; ↑246%
Chamras, Ardashian, Heber and Glaspy [37]	MCF-7	100 EPA	Diff-Quik Stain Set	120	NS Δ in % apoptotic cells
		100 DHA			NS Δ in % apoptotic cells

EPA = eicosapentaenoic acid; DHA = docosahexaenoic acid; PI = Propidium Iodide; TUNEL = Terminal deoxynucleotidyl transferase (TdT) dUTP Nick-End Labeling; ssDNA = single stranded DNA; NS Δ = no significant change; “↑” denotes significant increase (*p* < 0.05); “↓” denotes significant decrease (*p* < 0.05); “●” = statistical significance was not assessed; “%” = percent change from control condition; * Relative to control conditions.

**Table 11:** Change in Akt and pAkt with EPA:DHA mixtures in human BC cell lines.

Citation	Cell Line	Concentration of EPA or DHA (µM)	Assay	Exposure (Hours)	Effect on Akt and pAkt *
Schley, Jijon, Robinson and Field [29]	MDA-MB-231	60 EPA + 40 DHA	Western Blot	72	↓47% pAkt NS Δ in Akt
		45 EPA + 30 DHA + 75 LA			↓27% pAkt NS Δ in Akt

EPA = eicosapentaenoic acid; DHA = docosahexaenoic acid; “↓” denotes significant decrease (*p* < 0.05); NS Δ = no significant change; “%” = percent change from control condition; Akt = protein kinase B; pAkt = phosphorylated Akt; * Relative to control conditions.

**Table 12:** Comparison of EPA and DHA on Akt and pAkt in human BC cell lines.

Citation	Cell Line	Concentration of EPA or DHA (µM)	Assay	Exposure (Hours)	Effect on Akt and pAkt *
Cao, Ma, Rasenick, Yeh and Yu [23]	MCF-7	90 EPA	Western Blot	24	↓27% pAkt:Akt ●
		90 DHA			↓33% pAkt:Akt ●
Lee, Yun, Koo, Sung, Shim, Ye, Hong and Kim [25]	MDA-MB-231	30, 50 EPA	Western Blot **	24	NS Δ in Akt ●
		30, 50 DHA			↓Akt ●

EPA = eicosapentaenoic acid, DHA = docosahexaenoic acid; “↓” denotes significant decrease (*p* < 0.05); “●” = statistical significance was not assessed; NS Δ = no significant change “%” = percent change from control condition; Akt = protein kinase B; pAkt = phosphorylated Akt. * Relative to control conditions. ** Researchers did not quantify percent change in bands from control condition in Western Blot Analysis.

## Abbreviations

BC	Breast Cancer
n-3 LCPUFA	n-3 Long Chain Polyunsaturated Fatty Acids
DHA	Docosahexaenoic acid
EPA	Eicosapentaenoic acid
EGFR	Epidermal Growth Factor Receptor
PL	Phospholipid
ER	Estrogen Receptor
PR	Progesterone Receptor
HER2	Human Epidermal Growth Factor Receptor
FA	Fatty Acid
LA	Linoleic Acid
AA	Arachidonic Acid
PI3K/Akt	Phosphoinositide-3-kinase/protein kinase B
TNBC	Triple Negative Breast Cancer
